# Ableism in Occupational Therapy: A Critical Qualitative Analysis of Disabled Practitioners' Experiences in the Profession

**DOI:** 10.1155/oti/6295789

**Published:** 2025-11-06

**Authors:** Jenna L. Heffron, Kimberly J. The, Aster Harrison

**Affiliations:** ^1^Department of Occupational Therapy and Occupational Science, Towson University, Towson, Maryland, USA; ^2^Lurie Institute for Disability Policy, Brandeis University, Waltham, Massachusetts, USA; ^3^Centre d'Études et de Recherche sur les Services de Santé et la Qualité de Vie (CEReSS), Aix-Marseille Université, Marseille, France

**Keywords:** ableism, accessibility, accommodations, critical qualitative research, disability, disability studies, occupational therapy, productivity

## Abstract

**Purpose:**

The purpose of this study is to explore disabled occupational therapy practitioner (OTP) experiences of ableism in the occupational therapy profession.

**Materials and Methods:**

This study used a critical qualitative approach led by three disabled occupational therapists and informed by disability studies theory. Researchers conducted 12 semistructured interviews with disabled OTPs. Interviews were analyzed using codebook thematic analysis.

**Results:**

Experiences of ableism were pervasive for OTPs with disabilities. Disabled OTPs experienced ideological, institutional, interpersonal, and internalized ableism in the profession. Participants described varying views of the culture of occupational therapy regarding disability. Participants navigated their work through the use of social supports, formal, informal, and self-accommodations, and coping strategies. Disabled OTPs also described how their disabilities informed their approaches to practice, including antiableist and disability-affirming approaches. Disabled OTPs readily identified room for change in the profession and provided recommendations for improving accessibility and inclusivity in OT.

**Conclusions:**

Participants experienced disability oppression (ableism) in the profession. Structures and norms in the profession prioritized nondisabled ways of being that contributed to many of the barriers experienced. Participants' approaches and recommendations can help disrupt systems of ableism in occupational therapy.

## 1. Introduction

Occupational therapy (OT) is aimed at fostering meaningful participation, quality of life, and belonging for disabled people^1^. Occupational therapy practitioners (OTPs)^2^ are trained in assessment and intervention related to accessibility, disability accommodation, and workforce inclusion [1]. However, contrary to the profession's vision of being “intentionally inclusive and equitable and embrac[ing] diversity in all its forms” ([2], para. 4), disabled OTPs experience workforce discrimination and exclusion at interpersonal and institutional levels [3–6]. The growing body of literature on the experiences of disabled OTPs describes both overt and subtle interpersonal ableism, including treating disabled students like patients [7–9], expecting disabled students to mentor their disabled peers [10], supervising disabled practitioners in excess [11], equating disability with professional incompetence [6, 11], socially excluding and bullying disabled students and practitioners [4, 7, 11, 12], and discouraging development of disability identity [10, 13].

Institutional ableism is also well documented, including the bureaucratic and burdensome process of securing reasonable accommodations [4, 10, 12], rigid policies and programmatic inflexibility [4, 14], and exclusionary university processes [14, 15]. OT scholars advocating for antioppressive practices have emphasized the critical role of OT education and professional culture in shaping minoritized (including disabled) students' and practitioners' experiences and sense of belonging in the profession [16, 17]. Perhaps exemplifying disabled OTPs' experiences is the contradiction of being in a profession that champions inclusion for disabled people yet is often inaccessible and even hostile to disabled members of “its own flock” ([7], p. 590). In a systematic review that further validates the impact of discrimination against disabled healthcare practitioners, including OTPs, Lindsay et al. [5] reported a range of negative impacts of educational and workplace ableism on the physical and mental health, job satisfaction, and career trajectory of disabled practitioners.

While disabled OTPs experience a multitude of ableist barriers in their daily practice, they also use their strengths and experiences to make positive contributions across practice settings. For example, research has reported that disabled OTPs and students demonstrate critical reflexivity and use their dual identities to disrupt healthcare power differentials [7, 11]; build rapport with and provide better service to their clients [6, 14]; and model and advocate for antiableism in their setting and profession [6].

Ableism remains a pervasive force within OT, shaping professional norms and impacting professional and health outcomes for disabled practitioners. Critical OT scholars have urged the profession to engage with disability studies critiques [18, 19] and produce critical disability scholarship to resist perpetuating ableism in the profession [20]. Moreover, the recommendations of disabled OTPs are needed to ensure antiableism work is rooted in disabled practitioners' lived experiences and knowledge about ableism. The purpose of this study is to explore disabled OTPs' experiences in the profession using a critical qualitative research approach [21]. This study describes barriers disabled OTPs experience, supports they find helpful, and approaches that the profession could take to improve accessibility and confront ableism.

## 2. Methods

### 2.1. Design

This study used a critical qualitative research approach. Critical qualitative research is attentive to power relationships, systems of oppression, and their effects on the subject at hand and examines the structural and systemic root causes of social phenomena [21]. This study was designed with a disability studies [22] understanding of the power structure of ableism and its effects on OTPs and the profession of OT.

### 2.2. Research Team and Reflexivity

The authors all identify as disabled, are OTs, and have extensive experience working in disability communities. Two are white and one is Asian American. Two researchers are heterosexual women and one is bisexual and genderqueer. Critical qualitative research methods are aimed at questioning and disrupting social inequities in the research process. Disabled people are central to OT research, yet they are rarely afforded control over the research process and outcomes. In line with our critical qualitative approach, this research was led by three disabled OTs who also participated as interviewees to challenge researcher/participant binaries and ensure leadership by those most impacted by the research. The approach demanded continuous critical reflexivity [23]. Researchers engaged in reflexive conversations to intentionally consider participant perspectives that differed from the opinions of the three researcher–participants as reflected in our own interview transcripts and analyses. Researchers acknowledged and discussed our own preexisting experiences with the topics and theoretical frames and spent considerable time exploring the extent to which our analyses were grounded in the data. We were also informed by feminist methodologies of insider–outsider research in taking this approach (e.g., [24, 25]), including in our considerations of how to mindfully balance researcher and participant voices, “traveling between ‘speaking for' and ‘speaking from'” ([25], p. 131).

### 2.3. Data Collection

Participants (*N* = 12) were recruited via purposive and snowball sampling via social media and email. Upon receipt of participants' signed informed consent documents, interview data were collected via telephone for 11 participants and over Facebook Messenger for one deaf participant. Participants were in a variety of settings during their interviews, ranging from on their way to work to sitting at home. Interviews were completed either by the researchers or by students being supervised by the first author.

Each participant completed a semistructured interview lasting approximately 1 h. The interview guide was initially developed by the principal investigator (PI, first author) in collaboration with a team of students enrolled in a group research course taught by the PI. The interview guide was subsequently revised according to feedback from a key informant (second author). Interviews included questions about experiences in OT school and practice, accommodations, and recommendations for the profession. Participants selected their own pseudonyms, which are presented below. Interviews were audio recorded and transcribed verbatim. Field notes were also recorded.

### 2.4. Data Analysis

Data were analyzed using codebook thematic analysis^3^ [26, 27] and ATLAS.ti qualitative data analysis software Version 8.4.26 [28]. Braun and Clarke describe the six steps of thematic analysis as “(1) data familiarisation and writing familiarisation notes; (2) systematic data coding; (3) generating initial themes from coded and collated data; (4) developing and reviewing themes; (5) refining, defining and naming themes; and (6) writing the report” ([26], p. 331).

Three cohorts of 8–10 master's-level OT students had previously coded the data in their group research course, which assisted the researchers with data familiarization as they guided the students, although student codes were removed from the transcripts prior to recoding by the authors. The immersion of students in the research was part of the critical qualitative approach of this research, as student researchers' engagement with the data aimed to raise their consciousness about disability experiences and ableism. Students simultaneously engaged in educational sessions led by the researchers about disability research and disability studies as part of their research methods coursework. This study was approved by the Ithaca College Institutional Review Board (IRB 1116-13).

After the student research immersion and simultaneous researcher data familiarization, researchers reread the interviews and proceeded to write inductive memos about key patterns and ideas in the data. From these memos, we constructed a draft codebook through a process of team discussion, refinement, and revision. As we used this codebook to code the interviews, we had continuous meetings as a team to revise the codes, reconcile our usage of the codes, and discuss early notions of themes. Disagreements about code definitions and usage were addressed via extensive discussion and consensus. Each interview was first coded by a primary coder; then, codes were reviewed and verified by a secondary coder. Disagreements between the coders were resolved through discussion and consensus. After all interviews were coded, the team had a series of meetings to organize the codes into themes and subthemes. Negative case analysis [29] was used to balance participant voices in the creation of themes and subthemes. For example, if a concept emerged about a particular setting being especially inaccessible to participants, we searched the data to see if the opposite opinion was also represented. In such cases of disagreement among participants, the researchers discussed the possible explanations for the discordance and revised themes appropriately. The discussion of the pediatric setting in our results exemplifies this negative case analysis approach.

While the use of multiple coders, group discussion, reflexivity, and negative case analysis served to enhance trustworthiness that the themes were supported by the research data, codebook thematic analysis balances these considerations with acknowledgement of the importance of the researchers' unique interpretive perspectives. While the codes and themes are generated from the data, the researchers made sense of the themes through a disability studies lens. For example, multiple participants discussed experiences of needing to hide their disability status to appear nondisabled or less disabled. Our team labeled these experiences with the phrase *passing as nondisabled*, to connect these experiences to work in disability studies, critical race theory, and gender and women's studies about people from nondominant groups *passing as* members of dominant groups for safety. While these experiences would likely have still been highlighted by another team, they might have been described differently.

### 2.5. Participants

Due to participant concerns that they might be identifiable if we listed individual participant characteristics in a detailed table, this paper will only present demographic characteristics in aggregate. Participants identified with multiple types of disabilities including cognitive (41.7%), psychiatric (41.7%), physical (33.3%), sensory (16.7%), and other (16.7%). All participants were women^4^. They ranged in age from 24 to 41 years with a mean age of 30 years. Time in practice ranged from 5 months to 19 years with a mean of 5 years. They reported having disabilities for 2.17–34 years (mean = 11). Participants were from the United States (*n* = 9), Canada (*n* = 2), and Australia (*n* = 1). Nine participants were white. One participant identified as Asian, one as mixed race, and another declined to answer. They worked in a wide variety of settings including community, academic, and clinical settings. Half worked full time, 33% worked per diem, and 25% worked part-time.

## 3. Results

The theme of *ableism* was the most salient and foundational theme identified throughout and across interviews. *Ableism* informed and interwove with all other themes. This primary theme will be discussed in the greatest detail, followed by other themes including *supports and coping strategies* for navigating ableism in the profession*, antiableist and disability-affirming approaches to practice*, *professional culture in OT*, and *recommendations for change.* In keeping with the critical qualitative methodology of this research, the results explicitly engage issues of power and oppression. Disability studies theories are used to make sense of the findings about disability oppression (ableism). Given that some of these theories and concepts are not well known in OT, relevant literature is integrated in the results section where necessary to explain a theme.

### 3.1. Ableism

Disability oppression, or ableism, refers to a system that privileges nondisabled people and perspectives and perpetuates systematic, unequal treatment of disabled people on the basis of disability [30, 31]. Bell's [32] *four Is of oppression* (ideological, institutional, interpersonal, and internalized) describes oppression as a framework of four interrelated components and will be used to organize the ways in which participants experienced ableism in their OT educational and workplace environments.

#### 3.1.1. Ideological Ableism

Ideological ableism refers to long-held cultural ideas that able-bodiedness and able-mindedness are preferable to disability [32]. Sarah was describing ideological ableism when she said, “I think on an intellectual level, [OT practitioners] accept [disability], but I don't think on a real level they accept it...I think anytime there's someone with a disability, they speak to them a little differently.”

##### 3.1.1.1. Compulsory Able-Bodiedness

Participants' experiences with ideological ableism were often aligned with McRuer's [33] concept of compulsory able-bodiedness—an implicit or explicit imperative that practitioners (disabled or not) strive toward able-bodied and able-minded ideals. Rebecca describes some of the ways that compulsory able-bodiedness shows up in the profession:

I would say, especially in a [physical disability] setting, you have to be on your feet all the time. And that's difficult for anybody. I mean, even if you are in good shape, it's really, really strenuous. You have to be running around with patients all day, running back and forth getting supplies, running back and forth to get paperwork, back and forth to get this and you are lifting and transferring people all day. Even at the end of the day when you are totally done with the physical aspect of therapy, you have to sit at a computer for 30 minutes to an hour, maybe in excess of that, doing notes. All of that is just so fatiguing. It's a lot on your body.

Much of what Rebecca describes as the most exhausting aspects of her work day (e.g., retrieving supplies and materials and completing most or all of her documentation at the end of a long day) is not the skilled aspects of her job but rather those that could conceivably be reconfigured or delegated to balance workload, facilitate collaboration, and allow her to focus on the essential components of her role. Instead, nonessential job expectations, which are rooted in assumptions that OTPs are nondisabled and/or should strive to be, result in her working beyond her physical and time-related capacities.

Kay also spoke to the notion of compulsory able-bodiedness:

It's really hard because people do not usually think of healthcare professionals and then people with disabilities together. And I think it's because disability is seen as a bad thing whereas being a practitioner, like you have to be perfect, you have to meet all these demands, you have to be all these things. And so at first I came in with a lot of like, “oh yeah, I'll just assert myself and I have this identity and it's really important to me.” But I think as I went farther into the program, other people do not tend to see it like that. It's a lot of always me having to explain myself type of thing—it's really tiring.

Adrian explained that compulsory able-bodiedness showed up in the OT curriculum when all students were expected to participate as both the “patient” and the “practitioner” in role-playing. It was assumed that all students could perform normal range and strength for their classmates to practice manual muscle testing. When Adrian could not have fellow students complete manual muscle testing on her because of her impairment, her impairment was revealed to classmates.

#### 3.1.2. Institutional Ableism

Participants experienced barriers to entry, engagement, and advancement within both educational and workplace environments. In many cases, these barriers were caused by institutional ableism—the ways in which ableist ideologies become embedded into institutional processes and practices [32]. Participants in this study experienced institutional ableism in the form of rigid and normative infrastructure and an overly burdensome process for securing reasonable accommodations.

##### 3.1.2.1. Rigid and Normative Infrastructure

The infrastructure of OT education and practice, including formal requirements and unwritten rules about participation, was problematic for participants. Most participants' OT programs had a cohort-based structure. Students were required to complete a predetermined, sequential, full-time program with the same cohort from start to finish. For example, it is usually not permitted to progress through the program part-time, and if a student fails one course or needs to take a leave of absence, they are often required to take a year away from the program, retake the class, and then continue the program with the following year's cohort. Inflexible temporal demands, such as this lock-step, cohort-based structure (Hadassah, Sam) and inability to complete on a part-time basis (Hadassah, Sam, and Jackie) posed barriers for some participants. Sometimes these barriers followed participants into their careers. Sam explained, “I graduated later than the rest of my cohort … I guess it was a bit of isolation.” Several participants (Jo, Jackie, Rosie, Sam, and Hadassah) also cited the full-time nature of their programs as further exacerbating the financial burden of an already expensive educational experience. For example, Jackie took out student loans to pay for OT school because “there was no way I was going to function if I had to work and go to grad school [simultaneously].” Even so, by graduation Jackie was so exhausted that she needed a break prior to entering the workforce, which delayed her ability to pay back her student loans.

Rigid testing requirements limited access to the OT curriculum for participants, such as high-stakes lab practicums requiring performance in front of and often involving physical contact with peers or instructors (Rebecca, Adrian) and heavy emphasis on multiple-choice assessments that restricted some participants from expressing mastery of the material (Sarah, Rebecca). Sarah also noted that the doctoral field exam required by her postprofessional occupational therapy doctorate (OTD) program—intended to ensure adequate preparation for the doctoral capstone project—was inaccessible to her as someone with a learning disability, as it required extensive writing within a short timeframe, thereby demanding speed of productivity. Participants also reported challenges with the “horrible, horrible” (Rebecca) US national certification exam.

Normative expectations during OT fieldwork included limited availability of part-time placements (Sam) and long commutes—such as Kay's 2.5-h commute. Ableist workplace expectations included high productivity standards, which Rebecca, Adrian, and Jackie explained required them to put in more time and effort than their nondisabled peers. Participants also described leave policies as problematic. Max was experiencing an acute flare-up of her condition at the time she was supposed to begin her first OT job, but her employer was not willing to postpone her start date, so she was forced to work through the flare-up in order to maintain employment. This led to her being hospitalized and subsequently fired because her employer would not allow leave during the hospitalization. Jackie's quality of life was negatively impacted by her employer's paid time off policy that pools sick time and vacation time, forcing her to choose between reactively and proactively caring for her health: “I can't really take vacations because I should really be using them for sick days.”

##### 3.1.2.2. Acquiring Adequate Accommodations

All nine participants who had formal accommodations expressed concerns about the complicated and taxing process of securing them. Many institutions require a medical statement prior to providing accommodations. This process of “proving” disability led to unwanted medical encounters for Sarah and negative experiences of skepticism or stigma from medical professionals for Jackie. Several participants also described insufficient support from faculty, fieldwork coordinators, and disability services staff to navigate the (often diverse) demands and available accommodations across classroom, fieldwork, and work environments (Sam, Hadassah, and Kay). Kay explained, “every setting is different, and so I wish the fieldwork coordinator would have helped me translate that a little bit better.”

Even though accommodations and accessibility are well within OT's domain, many participants reported supervisor and educator ignorance about rights to accommodation. Sam reported that her OT school did not educate students about accommodations (for themselves or for clients), and Max encountered resistance to accommodations in her academic program. June and Adrian were pressured by their supervisors (both OTs) to disclose diagnoses before receiving accommodations at work or fieldwork—a practice which is not in keeping with legal protections afforded by the Americans with Disabilities Act [34].

#### 3.1.3. Interpersonal Ableism

Interpersonal ableism refers to inequitable treatment of disabled people that manifests on a social level between individuals, including via conversations, nonverbal approaches or gestures, and relationships. Interpersonal ableism is often either justified or reinforced by deep-rooted ideological and institutional ableism [32]. Participants experienced interpersonal ableism in various forms, including being presumed to be incompetent, feeling pressured to pass or forced to disclose disability, and being the object of a clinical gaze.

##### 3.1.3.1. Presuming Incompetence

Some participants described being on the receiving end of negative attitudes and low expectations from others based on their disabled status. These instances are consistent with Nario-Redmond's [35] explanation of presuming incompetence as a component of ableism. Applying this principle to the experiences of disabled OTs in this study, expectations and attitudes from peers and supervisors sometimes appeared to be based on the idea that disability makes someone inherently less competent. For example, Rebecca, June, and Adrian sometimes chose not to disclose disability nor use accommodations out of concern for being treated differently by their peers. Rebecca reported choosing to “struggle in silence” due to pressure to be seen as competent. Jackie expressed concern that while her current colleagues were supportive, “there's a fear of like if I leave this job I don't know how another place, how accepting they would be.”

Sometimes bias about disability was subtle, such as being discouraged from applying for a leadership position after disclosing disability to a supervisor (Sarah) and coworkers being impressed that they were not able to “tell” that the OT had a disability (Jo). In other cases, discriminatory viewpoints and actions were overt. For example, Adrian won a competitive fieldwork placement, yet when she submitted a letter of accommodation to her site supervisors, they retaliated, threatening to remove her from the placement and creating a hostile work environment throughout the remainder of the placement. In these examples, the belief in the superiority of nondisabled ways of being (ideological ableism) was intertwined with unsustainable productivity standards (institutional ableism). These forms of ableism shaped participants' experiences, such as being passed over for a promotion and facing attempted removal from a fieldwork site after coming out as disabled (interpersonal ableism). This led participants to hide their disability, not seek out needed supports, navigate unnecessarily toxic workplace environments, and experience barriers to career mobility and fulfilling their potential.

##### 3.1.3.2. Passing as Nondisabled and Forced Disclosure

Given the able-bodied and able-minded ideals that participants felt obligated to conform to, many felt inclined or forced to appear or *pass* as nondisabled, or as close to nondisabled as possible (Rosie, Adrian, Jo, June, Kay, Max, and Jackie). Samuels [36] explains that passing as nondisabled is a complex phenomenon, which can happen both voluntarily and involuntarily. Sometimes passing as a member of a normative group can afford safety, but it can also come at the expense of not receiving community recognition or needed resources. For participants in this study, passing as nondisabled was usually done to fit in with norms of compulsory able-bodiedness and avoid disability stigma and discrimination. For example, Rosie, an amputee, described her experience with strategically choosing when to pass as nondisabled:

It's always been very interesting because I could always view life from two different angles. So if I wanted to view life as a nondisabled person, I could go out with pants and no one would really know [about my amputation]. And if I went out wearing shorts it was a different experience.

Other participants had more apparent or visible impairments and had moments where they were not able to pass as nondisabled, impacting their future decisions about disclosure and/or efforts to pass (Max, Sam, Jackie, Kay, and Adrian). Kay's experience epitomizes the importance of passing as nondisabled as a safety tool. She said, “sometimes I struggle with making my disability more visible especially since I just went through an experience where clearly disability was not valued in the profession, so it's a little bit of a soft spot for me right now.” Kay grapples with the desire to publicly embrace her disability and what it brings to her therapy practice, yet her past experiences make it unsafe for her to do so. Jackie worries that disclosure would put her at risk at work because her employer would view her as less efficient or capable: “there's that fear of like it's still a professional environment and it's a business, they want to make money.” June and Adrian, as described earlier, were pressured to disclose their disabilities.

Participants navigated disclosure decisions based on the context and power dynamics at play but also based on perceived benefits to clients or families. Fran described disclosing her history with learning disabilities to parents to give them hope about potential progress in functional goals. She also used her disability to connect with pediatric clients: “Yeah, I'll be like ‘oh when I was a little girl, I had a really hard time cutting, cutting on the lines is really difficult.' Things like that to make them feel more comfortable...to relate better with that and to build rapport.”

##### 3.1.3.3. Clinical Gaze

Participants described experiencing a *clinical gaze* applied to them by peers and superiors. The term clinical gaze, adapted from Foucault's [37] term *medical gaze*, refers to the application of clinical reasoning to evaluate the experiences, perspectives, or performance of a disabled OT. When viewed through a clinical gaze, a disabled person is treated as an object of clinical analysis and assessed in biomedical terms, rather than being viewed holistically as a peer with a whole range of human experiences. Adrian described her experience of peers applying a clinical gaze to her in OT school:

My neuroatypicality and my mental illness, those are things I was extraordinarily cautious [about] the entire time I was in OT school to not disclose. Because I had disclosed to one or two people my first year and they would casually say something like, “Oh, do you think you're really excited or do you think you're just manic?” That sort of clinical gaze that you do not want from your friends, you just want your friend to just say, “That is amazing that you are excited about that,” not trying to diagnose you.

Upon Adrian's disclosure of disability, the OT students utilizing a clinical gaze were quick to overattribute Adrian's thoughts and emotions to disability and compare it to a set of symptoms classified with her disability.

Kay described also that colleagues would sometimes be too quick to overshare medical opinions about her diagnosis, rather than listening to her about how it manifests. “They'll suddenly pretend they know everything about brain injuries, when like they are probably gonna be wrong because my brain injury manifests itself really differently, and I'm really good at adapting and things”.

#### 3.1.4. Internalized Ableism

The final aspect of ableism that participants experienced was internalized ableism. This occurred when, over time, participants came to internalize the idea that disability is inferior to able-bodiedness and able-mindedness or even that they themselves are incompetent or need to overcome their disability in order to be a competent OT [32].

Internalized ableism was present across all participants' interviews. For example, Fran considered whether she had passed her impairment onto her children, stating, “knock on wood, I don't think any of them have a learning disability,” demonstrating that as a disabled person, she was not immune to internalizing the notion that a learning disability is a bad thing to have. Other participants described having a prosthesis as cooler than “having a deformed leg” (Rosie), referred to their accommodations as “special examination arrangements” (Sam), or otherwise used ableist terminology referring to themselves or other disabled people (Rosie, Kay, and June). These instances align with Bell's [32] description of internalized ableism. As members of an oppressed group, disabled OTPs may be made to feel badly about themselves yet may not have the power to direct those feelings back to the systems, structures, and people oppressing them. As a result, “there are only two places to dump those feelings—on oneself and on the people in the same group” ([32], p. 3). The former is apparent in Sam's response when asked to describe supports she had in OT school: “I guess because I really wasn't managing well so it was things like...a little bit of extra time [on exams].” Rather than describing accommodation as a legal right to level the playing field in an otherwise inaccessible curriculum, Sam blames herself, attributing her need for accommodation to personal struggle or ineffective coping. Further, she downplays her need for accommodation, suggesting an internalized belief that needing support is a sign of weakness or failure.

In reference to her work colleagues, Jo stated, “they really have been awesome about just kind of accepting me and being like ‘oh yeah, she's an amputee, but that doesn't stop her.'” On the surface, this may appear to be an instance of a disabled OT appreciating an inclusive workplace or supportive colleagues. However, it is also reflective of the ableist idea that “overcoming” disability should be celebrated, as well as Jo's internalizing of this negative message. In this instance, Jo demonstrates gratefulness that the profession simply includes her, even though she still experiences ableist barriers within it.

### 3.2. Supports and Coping Strategies for Navigating Ableism in the Profession

Participants identified a variety of supports that helped them navigate ableism in the profession, including social supports, formal accommodations, informal accommodations, and self-accommodations. Eleven of the 12 participants described the importance of social support from friends and/or family. Several participants reported social support from professional leadership, including supervisors (Rosie, June, Sam, and Jackie), teachers/faculty (Kay, Sam), or fieldwork coordinators (Sam, Adrian) who facilitated access to accommodations. Adrian explained, “thank goodness we had a fieldwork coordinator who knew something about disability rights and was willing to say [to the site threatening discriminatory dismissal], ‘you absolutely cannot treat a student this way because she is disabled.'” Other participants found social support from some of their coworkers (Jackie, Fran, Rosie, Rebecca, and June). Several participants (Jackie, Kay, Hadassah, Adrian, June, and Sam) emphasized the importance of disabled peers as a key resource for both social support and brainstorming accommodations for fieldwork and practice.

Many participants felt that a formal group for OTs with disabilities would be helpful, but few knew about such a group. One of the few who knew about a formal organization was June, who recommended involvement with the US-based Network for OT Practitioners with Disabilities and Supporters [38].

#### 3.2.1. Formal Accommodations and Supports

Despite barriers in the accommodation process, once formal accommodations were secured, they were an important support to many participants during OT education (Kay, Sam, Sarah, Max, Jackie, and Hadassah), fieldwork (Kay, Sam, and Adrian), and the US national certification exam (Jackie). Participants did not describe formal accommodations at work. Several participants said that knowing about disability rights and/or disability studies (Jo, Kay, Adrian, Sarah, and June) was helpful—particularly in advocating for accommodations (Kay, Adrian).

Some participants had to teach their OT school how to accommodate them, such as Max: “It was a lot of learning…they had to figure out how to caption videos and how my placements were gonna work. And the teachers had to learn how to teach with ASL interpreters.” Max explained that she picked her OT school not because they knew how to accommodate her but because they were the school that seemed most willing to learn how to accommodate her.

A few participants (Max, Sam, Hadassah, and Rosie) also reported that they received support from the federal government, such as through a disability employment program (Sam) or assistance with purchasing adaptive technology (Max). Rosie was the only US participant who reported support from a state program, and she also received support from a disability nonprofit.

#### 3.2.2. Informal Accommodations

Numerous participants (Jo, Sam, Max, Jackie, Sarah, Rosie, June, Fran, and Kay) had arranged informal accommodations—disability supports in their school or work that were casually arranged through conversation with a supervisor or coworker “out of favor” (Kay) but not formally documented as a legal right. For example, when Rosie returned to work after her amputation, she reached out to her supervisor about the possibility of wearing sneakers, which was a departure from typical attire in the setting:

It was telling my boss, “This is what I wanna do” and I think that was really all it was and she's like, “sure” [laughing]. And because it was so easy and did not impact them financially, it was pretty easy to be able to go ahead with it.

Some participants (June, Max, Jackie, and Sarah) described work or school settings that were either coincidentally accessible or may have been designed with access in mind, resulting in meeting the person's access needs without formal accommodation. For example, when a program offered enough time on exams (Sarah), flexibility with scheduling (Max, Jackie, and Sarah), and an office in a convenient and quiet location (June), participants were able to maintain access without navigating the accommodations process.

#### 3.2.3. Self-Accommodations and Coping Strategies

Many participants developed their own self-accommodations (Hadassah, Fran, Sarah, Jackie, Rebecca, June, and Max) instead of or in addition to relying on formal or informal accommodations. We defined *self-accommodations* as modifications to their jobs or workspaces that participants made themselves, rather than asking their colleagues, supervisors, or workplace to take care of these accommodations^5^. Self-accommodations included purchasing or acquiring adaptive equipment or assistive technology, taking on the role of educating others about accommodations, and selecting practice settings. Fran, Max, Kay, and Hadassah used adaptive technology to perform their duties as OT practitioners or students. Hadassah thought that her employer would not agree to purchase adaptive equipment due to the cost, so she purchased it herself. Max reported training and coaching her OT school instructors on how to properly work with her ASL interpreters. Many participants (Jackie, Rosie, Max, Hadassah, Jo, Fran, and Sarah) accommodated themselves by being strategic about which practice setting to work in. Some participants may have been interested in other settings but expected that the setting would not be made accessible to them. Instead, they strategically selected a more accessible practice setting.

*Coping strategies* were different from self-accommodations in that they did not represent the participant making an accommodation themselves that otherwise could have been made by their workplace. Rather, participants' use of coping strategies involved making changes to their routines, bodies, or selves off the clock to deal with a lack of accommodations or to avoid the process of securing accommodations. While better workplace or school accommodations could reduce the need for these coping strategies, they were not in themselves accommodations that could be offered formally or informally by a workplace or university. Coping strategies could be tiring, expensive, or painful. Max explained that she got cochlear implants after OT school because “there is no way I would have been able to find a job that would provide ASL interpreters for seeing the clients, it just wouldn't happen.” Jackie, Fran, Kay, Max, Rosie, Sam, and Sarah also mentioned putting in extra study hours, pursuing therapy, starting or changing medical treatments, exercise, and self-care strategies to perform better at work or school. Both *self-accommodations* and *coping strategies* represent the participant taking individual responsibility to make their job work for them, rather than asking for formal or informal workplace accommodations.

### 3.3. Disability-Affirming and Antiableist Approaches to Practice

Participants described how their personal experiences with disability change and improve the ways they practice. They further explained how their approaches to OT treatment aligned with OT values and could be utilized by other OTPs more broadly to advance the profession. Key approaches included radical client-centeredness (Sarah, Kay, Adrian, June, and Jo), peer support (Jo, Jackie, Rosie, and Hadassah), environmental accessibility (Sarah, Hadassah, June, Max, and Jackie), and advocacy (Rosie, Sarah, Kay, and June). Participants described practicing from the perspective that disability can be a strength rather than a hindrance (Jo, Fran, and Rebecca).

#### 3.3.1. Radical Client-Centeredness

Sarah, Kay, Adrian, and June spoke about radically client-centered approaches, often stemming from their experiences with disability. In this paper, we define radical client-centeredness as a practice of deeply valuing disabled lived experience, prioritizing self-direction of care, and facilitating greater autonomy than is usually afforded to clients in OT sessions. Examples of participants' radically client-centered approaches include disability rights advocacy (Sarah), community care (Kay), critical reflexivity about practitioner and professional biases (Adrian), and responsiveness to disability community critiques (June). Jo's client-centered approach—shaped by her own disability experience—is flexible and shares power with clients rather than prescribing a single “expert” way of doing things:

At the time of my amputation I was like, “there's no way I'm showering a way I don't want to shower or dressing a way that's cumbersome or not quick.” So now I'm really more focused on the patient and like, “Hey what do you need to shower?” “What situations do you have because I want to adapt to that.” I do not want to just tell you, “Oh you need a shower chair because you lost your leg,” that's not fair.

#### 3.3.2. Peer Support

Hadassah spoke to the distinct power of connecting with disabled peers for support rather than turning to colleagues or professional associations. If a barrier were to arise—such as difficulty during an assessment—she would turn to people she knew in the disability community, like members of a dedicated Facebook group, because “they would know better” how to help her navigate the situation. June expressed a similar perspective and added that being disabled enhanced her ability to couple professional and peer support when working with clients. Instead of approaching sessions solely with textbook and clinical knowledge, she combined academic expertise and lived experience to engage in creative problem-solving with clients. She emphasized that “being part of the disability community is this whole other level of what I can offer to clients, and to students, and to colleagues.” Rosie spoke positively about receiving peer mentorship from another amputee as she prepared for her amputation. She shared that having the opportunity to ask questions and connect with someone who had personally gone through the experience helped her feel more prepared for the transition. Her reflections highlight the potential value of peer mentorship in OT, particularly for people who are newly disabled.

#### 3.3.3. Environmental Accessibility

Participants focused heavily on creating accessible environments, both for themselves and their clients. This included modifications to their immediate surroundings as well as attention to broader institutional and societal-level factors (Sarah, Hadassah, and June). For example, Max described modifying her immediate educational environment by using accommodations throughout OT school. Similarly, Jackie, who was adjusting to a new disability, used accommodations to navigate temporal challenges and physical aspects of her environment:

Really the first time I used supports, since I was diagnosed with narcolepsy my last semester, I actually did get accommodations for the [US national certification exam] because I knew that tests were very difficult for me. I could not sit in the quiz for long periods of time without falling asleep, so I did end up getting accommodations with the [US national certification exam] but that was the only time I ever officially had supports. (Jackie)

Hadassah, Sarah, and June expressed a commitment to engaging with broader institutional and professional challenges. For example, Hadassah described her involvement on a task force aimed at improving accessibility within the profession, while Sarah shared that she helped launch a volunteer chapter of a national disability advocacy organization in her home state.

#### 3.3.4. Advocacy

Advocacy was another key approach, which involved recognizing a situation as unjust and working to change it. Disability rights advocacy was used to draw attention to the importance of policy affecting disabled people (Rosie, Sarah, and Hadassah). Advocacy was sometimes done on behalf of the client, such as advocating in order to get medical equipment covered by insurance (Hadassah, Kay). In other cases, participants used their own experiences to empower clients and equip them with tools for self-advocacy:

So, going out in public, going out to a playground with a family, talking about what it's like to have everyone stare at you and different things that you can say and ways that I've reacted to people and the ways that work out very well when I've done it. And also, just how to work with doctors and understand that you have a lot of choice… And if a decision's being made that you do not think should be, continue to look for the doctor that you feel confident [in]. (Rosie)

### 3.4. Professional Culture in OT

OT professional culture can be understood in terms of knowledge, assumptions, values, norms, and practices that are shared among practitioners and students in the profession [40, 41]. Professional culture is complex, ever evolving, and arguably difficult to define. Participants described various perceptions about professional culture in OT regarding disability. While participants agreed on several accounts, they also expressed significant differences. Diverging opinions could be the result of participants moving through different environments (e.g., one participant having a more affirming workplace) and/or participants' different demographic characteristics (e.g., disability type, race, and gender) and perspectives on the profession and ableism.

#### 3.4.1. Ableist Culture

Several participants (Kay, Adrian, Hadassah, and Sarah) described a perceived hypocrisy in the profession, which touts its members as champions of environmental modification and participation. Sarah elaborated:

It was very offensive to me because here we are occupational therapists and we should be thinking about how we can challenge the environment, or how we can be more supportive of people and accommodate for them so that they can thrive, and then the very profession that I am working in is not doing that for me. Which I found to be hurtful and disappointing.

Hadassah agreed, stating, “I think OT generally, you would think like, ‘Oh yeah, OTs are the most inclusive bunch' considering our philosophies and the foundation of our profession, but it's not always the case.”

Other participants (Kay, Adrian, and Rosie) expressed concerns about ableism within pediatric settings. Describing the discrimination experience in which her pediatric fieldwork setting did not want to accept her as a student once she presented her letter of accommodation, Adrian said:

To me that just really called into question a lot of beliefs that I held about the profession, because these are people that work with disabled people every day and they somehow have such a hatred or discrimination against disabled people. They hate—they did not want to have a student that is disabled, even if she was meeting expectations. So, that really made me feel like, “What is this? Why are OTs, how are OTs working with disabled people every day if they do not even want to have one of them as a colleague or a student?”

Here and across other themes, participants sometimes reported vastly different opinions and perspectives about the culture of the profession. Six of the 12 participants mentioned that the culture of OT was more accepting than other professional cultures, including business (Jackie), accounting (Rosie), physical therapy (Kay and June), medicine (Kay and June), and speech therapy (June). Although participants differed in the extent to which they defined the *professional culture in OT* as ableist, all participants had experienced ableism within the profession.

#### 3.4.2. Affirming Culture

Several participants felt that the climate of the profession was positive toward them as disabled OTs and stated that they found fellow OT practitioners to be helpful and understanding. Jo explains:

I think it's actually a really great field to be in with a disability...I have not been to a setting where I feel uncomfortable or you know pushed aside or been like, “oh you know you can't do that because you have an amputation.” Everybody has been so awesome! Because they all just want to help.

In this instance, Jo describes her colleagues' explicit attitudes toward disability as welcoming and supportive. While Kay and Adrian experienced intense ableism in pediatric settings, several other participants felt that the climate of pediatrics was particularly affirming, including Fran, who stated:

I have no idea of what OTs in subacute or acute or hand care are like. The climate of OT pediatrics, they are playful. They are there to help children and they are working with children every single day so their mentality is different than maybe you would see in other places.

Several felt that the culture regarding disability really depends on the particular workplace or team. Jackie felt her current colleagues were supportive yet she was unsure about how affirming other settings would be, which put her in a difficult position given that she did not want to stay in her current position for much longer:

So it's kinda scary to really think about in the future, when you start a job, because then you have to interview and you are like, “I can't work 40 hours,” but how do I tell this person? You know what I mean. It's scary to think about.

Max echoed these sentiments, stating, “it really depends on the culture of the team or faculty.”

##### 3.4.2.1. Valued Aspects

Participants appreciated OT's grounding in a holistic approach, psychosocial focus, and empathy, as well as its focus on participation and environmental transformation. Participants often described these components as reasons that they entered the profession or reasons they prefer the profession over others. These valued aspects provide a foundation to build upon when looking toward more accessible futures for disabled OTs (Sarah, Hadassah, Kay, Adrian, and June). Participants valued what flexibility they had in their current work settings and wished for their colleagues to take a more creative, collective approach to ensuring workplace accessibility for everyone, including disabled OTPs (Max, Jackie, Rosie, Sam, and Hadassah).

### 3.5. Participant Recommendations

Participants shared ideas for improving accessibility in the OT profession based on their experiences. They often approached inaccessibility from the mindset that the profession could improve and identified recommendations for OT education, national leadership, workplaces, and an overall paradigm shift in the profession.

#### 3.5.1. “Room for Change”

Participants identified many areas within the profession where they saw “room for change” (Kay). Sam stated that even if the profession is inherently accepting of disabled practitioners, “I don't think the message is loud enough that it's okay and we seek it and we value it, or...like we want practitioners who have lived experience. Who have different and new ways to problem solve.”

Rebecca saw potential that is unique to this field:

I think it really gives us a unique spin on things because we do have education in the whole spectrum of disability. You know, the psychosocial aspect and the physical aspect and how they are interrelated. I really think it does give us an important perspective in terms of what we see.

Participants discussed a need for a cultural shift that would make disabled OTPs feel safer to disclose disability. They explained that such a culture would value the expertise of disabled OTPs because of, not despite, their disabilities. Kay emphasized the potential for practitioners to show up as allies to the disability rights movement organizing events to demonstrate solidarity with disabled OTPs and clients.

Rosie shared that “some therapists I've worked with...can maybe get very stuck” on normalizing the quality of children's movement where they could instead focus on functional movement. Kay felt that professional culture toward disabled practitioners is linked to how the profession views patients:

So, it's just like why are we imposing this idea of normal, and like I'm definitely not that idea of normal. So like, I know you are viewing me as a person with a disability, I know that translates to the clients and I know that it's teaching them something about like, that disability is inherently bad, and so we *need* to try and correct that. (Kay)

Adrian agreed, pointing to the possibility for the profession to grow in its antiableism through examining disabled practitioners' experiences:

The way that OTs with disabilities are treated really shines a light on some of the ableism within our profession…If we could improve the way that our profession treats OTs with disabilities that would really improve the way that our profession treats patients…It's the same, it is two sides of the same coin of disability oppression.

#### 3.5.2. Recommendations: Education and National Leadership

Participants recommended taking steps to improve the financial accessibility of OT education, integrating disability-affirming content and inclusive teaching methods, supporting disabled students and professionals, and advocating for systemic changes that promote disability rights and representation. Participant recommendations for OT education and national leadership are summarized in Table 1.

#### 3.5.3. Recommendations: Work Settings

Sam and June suggested workplaces could provide mentorship for disabled OTPs, and Rebecca advocated for more understanding toward coworkers having difficulties. Adrian and Kay suggested organizations emphasize interdependence and teamwork. For example, teams could divide tasks based on strengths so that a practitioner who had difficulty with one task (e.g., transferring) could trade with a coworker and spend more time on a task that matches their strengths (e.g., educating). Adrian also suggested that workplaces could take more of a team approach to productivity—valuing each person's contributions instead of fostering competition about treatment units and documentation speed. Adrian pointed out that critically re-evaluating the essential functions of OTPs is also important in supporting practitioners with disabilities:

We get really caught up in, “Oh, but can you work an 8-hour day? Can you do things really quickly? Can you transfer people by yourself?” When I do not actually think that is the most important part of being an OT. I actually think that being able to look at a person holistically and design something that works for them is the most important part.

#### 3.5.4. Recommendations: Paradigm Shift

Participants emphasized the importance of a cultural and paradigm shift in the way the profession thinks about disability. Participants wanted to see the profession prioritize disabled expertise in the same way it does professional expertise. Kay, June, Jackie, and Max explained that the profession needs to change attitudes around disability so that practitioners will feel safer to disclose their disabilities. They felt that raising awareness that both clients *and* practitioners may have disabilities would be key to creating a more supportive climate. Kay explained that the profession needs to “[break] down this divide between person with a disability and therapist because we can be both.” June emphasized taking action:

We cannot just be talking about it, we need to really take concrete steps to create change and to be in this continuous dialogue with practitioners with disabilities, with the disability community, to not assume what they want… if we are saying that we are client-centered and… holistic and care about meaningful participation in society, this stuff matters and we need to really make a concerted effort to make good on our values and to really translate our values into action.

## 4. Discussion

Consistent with prior research (e.g., [5, 7]), participants in the current study reported experiencing ableism at institutional and interpersonal levels. This study also expands the investigation of ableism by elucidating less visible forms, including ideological and internalized ableism. It is important to note that while all participants experienced ableism in the OT profession, they did not all use the word ableism to describe these experiences. It is possible that some participants were unfamiliar with the term ableism or were reluctant to use the term to critique a profession that they are trying to assimilate within. Given their immersion in a profession that generally expects professionals to act in a neurotypical and nondisabled manner, participants may have internalized the challenges they experience as being a result of their own deficits rather than barriers within the professional environment. This self-blaming is common for practitioners who are trying to reconcile their own experiences of discrimination or exclusion in a profession that prides itself on being inclusive [7, 11, 13]. Still, regardless of whether participants used the term ableism when sharing their experiences, they all described instances of prejudice, discrimination, and/or exclusion on the basis of disability.

### 4.1. On Productivity

Participants were negatively impacted by profit-driven efficiency and productivity expectations in ways that mirror concerns reported elsewhere in the literature. Sometimes, the mechanisms that workers use to maintain their health and work-life balance were not sufficient supports for the participants. For example, Max's employer did not allow leave during the first weeks of her job, so when her start date coincided with a flare-up of her condition she was forced to work through the flare-up, which led to an acute crisis situation and resulted in her hospitalization. To make matters worse, Max was effectively fired for being hospitalized (and thus needing to take leave during the no-leave window), even though her hospitalization was caused in large part by the leave policy in the first place. Had Max been allowed to take time off when she needed it, she may have been able to return to work once her flare-up had passed. Instead, she was disabled by her employer's rigid leave policy that left her permanently unable to work there and that caused her human pain and suffering in the name of efficiency and profit. This example illustrates how labor relations under capitalism “create (and then oppress) the so-called disabled body[mind]” ([42], p. 2).

Unrealistic healthcare productivity standards have been shown to lead to negative outcomes for both practitioners and clients, including practitioner burnout and decreased quality of care [43–46]. In a profit-driven healthcare system, both disabled and nondisabled healthcare practitioners are required to sacrifice their own health and well-being to meet productivity standards, as demonstrated in Max's case. This imperative of efficiency and related burnout can interfere with practitioners' ability to practice with empathy and do their best clinical work [47–49]. Research demonstrates that practitioners who are burned out are more likely to make biased decisions and actions and engage in discriminatory care, thereby exacerbating inequities [46, 50, 51].

### 4.2. On Accommodations

Some participants secured formal accommodations to help them meet productivity expectations during OT school, including extended deadlines (Kay), extra time on classroom exams (Sarah, Kay, and Hadassah) and the US national certification exam (Jackie), extra time for fieldwork documentation (Kay), and reduced caseload during fieldwork (Kay). Formal accommodations are often referred to as “reasonable accommodations,” language that originates from the Americans with Disabilities Act. The use of the term “reasonable” is often used to uphold ableism through the justification that it is inconvenient for the organization or institution to make accommodations [52]. While essential for facilitating access in ableist and inaccessible systems, reasonable accommodations “do not challenge the system of capitalist white supremacy in which we are enmeshed” ([53], para. 22). Price [52] takes this argument one step further, asserting that not only do accommodations fail to challenge unjust systems, they “ultimately take us further away from any sustainable form of justice” (p. 112).

Although accommodations may be necessary to remedy immediate and concrete problems such as the structure of an exam, it is also crucial that the OT profession examines and reflects on what causes these barriers in the first place. Accommodations are reactionary in that they modify policy or procedure to mitigate inaccessibility when identified [54]. This approach to access is fragile, particularly in times of political instability and attacks on antidiscrimination legislation that afford rights to access (such as those occurring in the United States at the time of this writing). Rather than placing the onus on disabled practitioners to fit into a problematic and oppressive productivity model, a fundamental reimagining of systems would better mitigate practitioner burnout and facilitate better quality of care. In line with Kay and Adrian's recommendations regarding interdependent, team-based approaches, burnout caused by unrealistic workload and productivity expectations can be addressed through careful reconsideration of these expectations, flexible scheduling, creation of mutually beneficial support networks, and other policy changes at the level of institutions and systems [55–58]. Further, these approaches may translate to interventions and client care in ways that honor disabled people's wisdom, embrace disabled ways of being, and prioritize creativity and care over productivity and profit—something that disabled communities have been calling for [59, 60].

### 4.3. Where Productivity and Accommodations Meet

Healthcare and academic systems rely on competition to maximize profit, with professional competence assessed by measures of productivity and efficiency [55]. Thus, it makes sense that disabled OT practitioners, like many in this study, seek out accommodations to approximate what is deemed “normal” productivity by capitalist standards. Additionally, many participants also established *self-accommodations* to improve their ability to meet school or job expectations and remain competitive. The choice to pursue self-accommodations (like buying adaptive equipment to perform one's job which otherwise could have been purchased by the employer as part of a formal accommodation) can be seen as representing a rational rejection of the formal accommodation process which can be “futile” and “humiliating” ([39], p. 1) with its requests for medical documentation, long waits, and exposure to stigma. Relatedly, participants also resorted to modifying their own bodies (e.g., Max's cochlear implants) and lives outside of work to deal with the physical effects of inaccessibility and/or to continue to perform in the face of inaccessibility. These efforts have been described in the literature as “personal sacrifice” ([56], p. 8) and overcompensation “to reduce or minimize any devaluing characteristics” ([30], p. 23). Reliance on self-accommodations and personal coping skills may also indicate a desire, or perhaps even a need, to pass [36] as nondisabled to succeed in the profession. Disabled people often fear that they will be viewed as incompetent [3, 6, 11] or as a liability [7, 55] if they are out about their disability(ies). Indeed, participants in this study explained that they feared negative attitudes or other consequences if they were outed as disabled. Passing as nondisabled is a valid and often necessary strategy for surviving in and navigating ableist systems. However, it is also a product of a capitalist society that commodifies labor and privileges bodies and minds that minimize cost and maximize profit [61]. This capitalist system encourages workers to “overcome” their bodily differences through personal responsibility, such as self-accommodations and coping strategies, rather than ensuring the existence of a functional, humane workplace accommodation system. A capitalist and ableist healthcare workforce also pressures practitioners to appear as nondisabled as possible and to distance themselves from one another so as not to appear incompetent by association. This approach actively promotes separation between and within groups of disabled practitioners and creates a culture of silence [30, 62], thus hindering the ability to build community and confront ableism at all levels.

### 4.4. Disabled OTs Forging a Path Forward

The wisdom of disabled OTPs can change the ways that the profession provides care. Disabled practitioners can model reflexive insight, challenge dominant cultural notions such as independence and individualism, and leverage peer support and team-based approaches to advance the profession [11, 62, 63]. In the current study, participants shared recommendations such as re-examining ableist “essential functions” and productivity standards and questioning professional norms of competition and compulsory able-bodiedness. Participants also recommended prioritizing disabled perspectives and allying with disabled communities as ways for the profession to grow in its antiableism. These recommendations echo calls to action made by other disability studies–aligned OT scholars to resist ableist temporal expectations [64], confront the false binary of disabled client—nondisabled practitioner [20], apply systems-level analysis to concerns identified by disabled people [19], and name and contest oppression within OT and occupational science [65, 66]. While participants in the current study did not specifically recommend the dismantling of ableist educational practices and rebuilding curricula through the selection and implementation of antiableist frameworks [16, 65], they discussed the negative effects that ableist curricular content and processes had on them.

Participants' insights strengthen ongoing calls to action by revealing how ableism operates across ideological, institutional, interpersonal, and internalized levels. Their recommendations also reflect how the adoption and acceptance of key values of disability culture [67], such as interdependence and flexibility, could improve access for disabled OTPs and clients alike. Finally, approaches focused on client-centeredness, advocacy, participation, and environmental modification can be useful in moving the profession toward better alignment with disability community priorities of systemic and environmental change [68]. Our results suggest that disabled OTPs can lead the profession toward a more antiableist mission that interrogates bias and authentically partners with disabled communities [69–73].

### 4.5. Limitations

OT students interviewed some of the participants, which may have impacted the quality of the data collected, although they were supervised by the first author in their work. OT assistants were not included in the sample. Although the analysis was completed by three participants (the coauthors), results were not member-checked with other participants due to time and funding constraints. Participants were not specifically asked about how their other marginalized positionalities (e.g., queerness and racialization) impacted their experiences as disabled OTs. Future studies should take an explicitly intersectional approach (see [74]). Future research could also move toward testing the effectiveness of the antiableism recommendations included in this article, including via participatory approaches that directly involve disabled OTPs.

## 5. Conclusion

Despite deep-rooted and pervasive ableism in the profession, OT's core values can be mobilized to create a more just world for disabled people, including practitioners. Ableism often gets in the way of OT's values of participation, self-determination, and equitable access to meaningful occupation. Critically examining ableism in the profession, as well as the capitalist values and practices that support it, is crucial to creating a more just profession that is better able to meet the needs of a diverse society. Disabled practitioners' experiences illuminate structures of ableism in OT, and their leadership can help move the profession toward an antiableist future.

## Figures and Tables

**Table 1:** Participant recommendations for OT education and national leadership.

**Recommendation**	**Participant(s)**
Education	
Improve affordability of OT school	Rosie
Educate students about accommodations, disability, ableism, disability culture, and disability rights	Sam, June, Adrian
Teach about disability and accommodations as relevant both to disabled students/practitioners and clients	Sam, June, Adrian
Utilize multimodal teaching approaches for different learning styles	Sarah
Recruit, support, and value disabled students	Adrian, June, Sam, Max
Prioritize environmental interventions	June
Facilitate access to academic leadership positions for disabled people	June
National leadership	
Modify accreditation standards to require education on disability rights and/or disability studies	Kay, Adrian
Engage in national advocacy in partnership with disability communities and organizations	Kay, Adrian
Facilitate access to national leadership positions for disabled people	June
Amplify the presence of support groups and facilitate mentorship opportunities for disabled people	Jackie, Sam
Publish public, national statements recognizing disabled OTPs as a valued part of the workforce	Kay, Hadassah, Max, Adrian, Sam

## Data Availability

The data from this study are not publicly available in order to protect participant confidentiality.

## Endnotes

^1^In this paper, we use both identity-first (“disabled person”) and person-first (“person with disability”) language as both are used in disability communities and by our participants. “Disabled person” is often favored by critically conscious disability communities and is thus used more often to align with the critical approach of the research.

^2^The term *occupational therapy practitioners* (*OTPs*) is used in this paper when referring to both occupational therapists and occupational therapy assistants. The term *occupational therapists* (*OTs*) is used when referring to occupational therapists only, such as in the case of study participants, none of whom were occupational therapy assistants.

^3^Codebook thematic analysis strikes a balance between deductive and inductive approaches to qualitative research—codes and themes are generated interpretively from the data (not predetermined before analysis), and the role of the researcher as an interpreter with theoretical and experiential lenses is acknowledged. However, codes are developed relatively early and a codebook is constructed, permitting coding by multiple coders [26]. While interrater reliability was not calculated, discussion and agreement between multiple coders were used.

^4^The coresearcher who is genderqueer identified as a woman and used she/her pronouns at the time of the study, so is referred to in the data set in that way. This decision also serves to protect their anonymity when quoted.

^5^Our development of the *self-accommodation* theme concluded before the publication of Macfarlane's article about self-accommodation [39], and our definition differs somewhat from Macfarlane's. We differentiate between informally asking a coworker or supervisor for an accommodation (*informal accommodation*) and carrying out an accommodation independently without help of others at the workplace (*self-accommodation)*. Macfarlane's framework [39] would define both our concepts of *informal accommodation* and *self-accommodation* as self-accommodations. This is discussed more in the discussion section.
